# Dissecting Rice Polyamine Metabolism under Controlled Long-Term Drought Stress

**DOI:** 10.1371/journal.pone.0060325

**Published:** 2013-04-08

**Authors:** Phuc Thi Do, Thomas Degenkolbe, Alexander Erban, Arnd G. Heyer, Joachim Kopka, Karin I. Köhl, Dirk K. Hincha, Ellen Zuther

**Affiliations:** 1 Max-Planck-Institut für Molekulare Pflanzenphysiologie, Potsdam, Germany; 2 Universität Stuttgart, Biologisches Institut, Abteilung Botanik, Stuttgart, Germany; University of Delhi South Campus, India

## Abstract

A selection of 21 rice cultivars (*Oryza sativa* L. ssp. *indica* and *japonica*) was characterized under moderate long-term drought stress by comprehensive physiological analyses and determination of the contents of polyamines and selected metabolites directly related to polyamine metabolism. To investigate the potential regulation of polyamine biosynthesis at the transcriptional level, the expression of 21 genes encoding enzymes involved in these pathways were analyzed by qRT-PCR. Analysis of the genomic loci revealed that 11 of these genes were located in drought-related QTL regions, in agreement with a proposed role of polyamine metabolism in rice drought tolerance. The cultivars differed widely in their drought tolerance and parameters such as biomass and photosynthetic quantum yield were significantly affected by drought treatment. Under optimal irrigation free putrescine was the predominant polyamine followed by free spermidine and spermine. When exposed to drought putrescine levels decreased markedly and spermine became predominant in all cultivars. There were no correlations between polyamine contents and drought tolerance. GC-MS analysis revealed drought-induced changes of the levels of ornithine/arginine (substrate), substrates of polyamine synthesis, proline, product of a competing pathway and GABA, a potential degradation product. Gene expression analysis indicated that ADC-dependent polyamine biosynthesis responded much more strongly to drought than the ODC-dependent pathway. Nevertheless the fold change in transcript abundance of ODC1 under drought stress was linearly correlated with the drought tolerance of the cultivars. Combining metabolite and gene expression data, we propose a model of the coordinate adjustment of polyamine biosynthesis for the accumulation of spermine under drought conditions.

## Introduction

Rice (*Oryza sativa* L.) is one of the most important food crops in the world. Almost half of the worlds population depend on rice as their staple food [1]. Rice is particularly susceptible to soil water deficit [2]–[4]. For upland rice drought is a major constraint on productivity [5] and for rain fed lowland rice drought is the major environmental factor with a reduction of productivity up to 35% [6]. Most high-yielding rice cultivars developed for irrigated conditions are highly susceptible to drought stress as well [4]. The estimated average annual loss of rice production due to drought conditions world-wide is about 18 million tons, or 3.6 billion US$ [7]. Drought delays the development of the rice plant [8], and strongly affects morphology [9]–[11] as well as physiological processes like transpiration, photosynthesis, respiration and translocation of assimilates to the grain [12], [13]. Leaf and root phenology of rice cultivars are known to influence their vegetative response to water deficit [14]. The development of drought tolerant rice varieties is one of the challenges of the next decades (see [15]–[20] for reviews).

Polyamines, especially putrescine (Put), spermidine (Spd) and spermine (Spm) have been implicated in a wide range of biological processes, including growth, development and apoptosis [21]–[25]. Polyamines are also associated with responses of plants to environmental stresses, including mineral nutrient deficiencies, osmotic and drought stress, salinity, heat, chilling, hypoxia and environmental pollutants (for recent reviews see [26]–[28]. Treatment with inhibitors of polyamine biosynthesis reduces stress tolerance whereas addition of exogenous polyamines restores successful stress acclimation [29]–[31]. Therefore, polyamines are thought to play an essential role in the environmental stress tolerance of plants.

However, the physiological function of polyamines under abiotic stress conditions is not clear [32], [33]. Polyamines are positively charged at physiological pH and are therefore able to interact with negatively charged molecules, such as nucleic acids, acidic phospholipids, proteins and cell wall components such as pectin [34]–[36]. The multiple suggested roles of polyamines encompass involvement in protein phosphorylation, conformational transition of DNA [36], maintenance of ion balance, prevention of senescence, radical scavenging, membrane stabilization [34] and regulation of gene expression by enhancing the DNA-binding activity of transcription factors [37].

Also the specific functions of the different polyamines are unclear. Since Flores and co-authors reported massive accumulation of Put in leaf cells and protoplasts of oat in response to osmotic stress [38], a similar increase has been shown under osmotic or drought stress in rice [39] and other plant species [40]–[44]. Also, Spd and Spm accumulate under osmotic stress in some plants [31], [45], while a reduction of Put or Spd levels was observed in others [42], [44], [46], [47]. In more detailed analyses of rice Put, Spd and Spm were found to accumulate under osmotic stress [32] depending on stress intensity and duration [48].

Put is synthesized either directly from ornithine by ornithine decarboxylase (ODC; EC 4.1.1.17) or indirectly from arginine via agmatine. The pathway is initiated by the arginine decarboxylase reaction (ADC; EC 4.1.1.19). Agmatine is sequentially converted to N-carbamoylputrescine by agmatine iminohydrolase (AIH; EC 3.5.3.12) and finally to Put by N-carbamoylputrescine amidohydrolase (CPA; EC 3.5.1.53). Spd and Spm are synthesized from Put by the transfer of aminopropyl groups from decarboxylated S-adenosylmethionine (SAM). These reactions are catalysed by Spd synthase (SPD; EC 2.5.1.16) and Spm synthase (SPM; EC 2.5.1.22). The decarboxylated SAM precursor is produced from SAM by S-adenosylmethionine decarboxylase (SAMDC; EC 4.1.1.50). The details of polyamine biosynthesis have been reviewed (see e.g. [35], [36], [49], [50] for reviews). An integration of polyamine metabolic pathways into the surrounding metabolic network has been recently published for Arabidopsis [51].

The accumulation of Put during drought stress is thought to be primarily the result of increased ADC activity that may be controlled by transcript levels and/or enzyme activity in Arabidopsis [52], [53], rice [39] and other species. Abiotic stress tolerance of plants was improved by constitutive over-expression of various genes encoding polyamine biosynthesis enzymes. Transgenic approaches have recently been reviewed [27], [54], [55].

In this study we investigated changes in polyamine content in a wide range of rice cultivars after classifying them for drought tolerance after a mild long-term drought treatment. We analysed the transcript levels of all genes encoding enzymes involved in polyamine biosynthesis known in rice and quantified polyamine levels and changes in the pool sizes of metabolites involved in polyamine metabolism.

## Results

### Performance of the Cultivars

Natural genetic variation of rice cultivars was used to investigate the performance of rice under drought stress conditions. 17 cultivars from a Vietnamese breeding program for drought stress resistance were combined with four well characterized cultivars from the IRRI representing *japonica* and *indica* subspecies (Table 1). Subspecies of Vietnamese varieties was determined with six subspecies-specific sequence tagged sites (STS) markers, resulting in four *japonica*, eight *indica*, four *japonica* cultivars with *indica* introgressions and two *indica* cultivars with *japonica* introgressions (Degenkolbe et al. submitted).

**Table 1:** Cultivars of *Oryza sativa* L. used for moderate long-term drought stress experiments.

Cultivar	Number	Subspecies	Origin
CR203	1	*indica*	IBT
DR2	2	*indica*	IBT
Loc	3	*japonica/indica*	IBT
C70	4	*indica*	IBT
C71	5	*indica*	IBT
K. lua nuong	13	*japonica/indica*	IBT
Cuom	14	*indica/japonica*	IBT
Khau cham	15	*japonica/indica*	IBT
Khau hom	16	*japonica*	IBT
Khua non	17	*japonica/indica*	IBT
Tra linh	18	*indica/japonica*	IBT
Nep men	19	*indica*	IBT
Loc dau	20	*indica*	IBT
Lua man	22	*indica*	IBT
LC-93-1	29	*japonica*	IBT
LC-93-2	30	*indica*	IBT
LC-93-4	31	*japonica*	IBT
Nipponbare	50	*japonica*	IRRI
Taipei 309	51	*japonica*	IRRI
IR57311-95-2-3	52	*indica*	IRRI
Zhonghua	53	*japonica*	IRRI

For genotyping of the Vietnamese cultivars see Degenkolbe et al. (submitted). IBT - Institute of Biotechnology, Hanoi, Vietnam, IRRI - International Rice Research Institute, Manila, Philippines.

**Figure 1:**
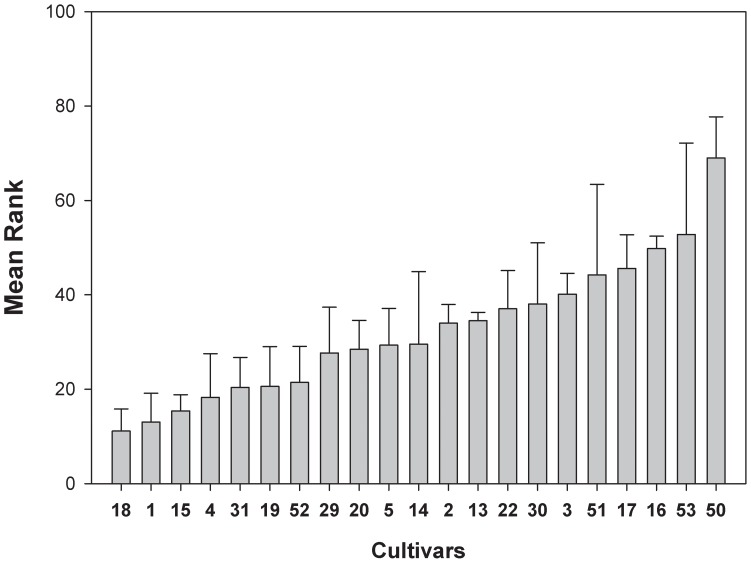
Mean rank of stress damage evaluated by visual scoring after 18 days of drought treatment. Each value represents the mean rank (±SE) of three experiments with five replicates each. Score of control plants was 1 (no damage) for 94% of the plants. Cultivars are ordered by mean rank. For cultivar numbers see Table 1.

The performance of the cultivars under drought stress was assessed during and after 18 days of stress treatment (Fig. S1). The drought stress score, based on the IRRI drought scoring scheme with a nine-level scale, was the most reproducible tolerance parameter between experiments and was thus used as the primary parameter for tolerance assessment. The cultivars varied from very tolerant to highly sensitive (Fig. 1). Shoot water content, predawn- and midday-leaf water potentials were significantly decreased after prolonged drought stress compared to control conditions, whereas only small changes were observed in the water content of the leaf blades (data not shown). All three parameters showed no correlation with the drought tolerance score.

Secondary performance parameters, such as biomass and photosynthetic quantum yield, were highly significantly affected by drought treatment (Table 2). The cultivars differed significantly in fresh and dry weight of the shoot and of the entire plant. Likewise, water use efficiency (WUE) under drought stress was significantly different between cultivars. Correlation analysis between WUE, PAM, yield, biomass parameters and drought score revealed a significant correlation between the score and the shoot dry weight (Table 2).

**Table 2:** Effect of drought treatment on performance parameters and their correlation with the drought score rank.

Parameter	Unit	Mean (Control)	Mean (Drought)	F (Condition)	F (Cultivar)	Correlation (neg. Score)
Score	–		33	–	*2.1*	1
WUE	g H_2_O/(g DW d)		0.056	–	***4.3***	0.15
PAM Yield	–	0.68	0.61	***47.1***	0.6	0.11
FW (Shoot)	g/Plant	16.2	2.7	***516***	***6.4***	0.19
DW (Shoot)	g/Plant	2.63	0.68	***275.4***	***4.5***	0.35
FW (Plant)	g/Plant	25.5	3.6	***396.2***	***5.2***	0.13*
DW (Plant)	g/Plant	3.34	0.82	***272.9***	***4.4***	0.26
Relative DW (Shoot)	–	1	0.29	–	0.03	0.16
Relative DW (Plant)	–	1	0.28	–	0.03	0.1

Mean values for the performance parameters water use efficiency (WUE), chlorophyll fluorescence (PAM Yield) 18 days after the start of the treatment, fresh weight (FW) and dry weight (DW) of the shoot and the entire plant, and relative dry weight compared to the control. F indicates the effect of the factor condition or cultivar on performance parameters. Bold and italic print indicates a significant effect with p<0.001, italic print p<0.05. Correlation (neg. Score) is the Spearman correlation coefficient between negative scoring rank and the performance parameters, significant correlation (p<0.05) are indicated by an asterisk (*).

### Polyamine Metabolism-related Metabolite Content of Rice Changes during Drought Stress

Changes in the pool sizes of the three predominant polyamines during drought stress were analyzed in the leaves of the selected rice cultivars. Polyamines can occur as free bases, as conjugates to small molecules such as phenolic acids (conjugated forms) and as conjugates to macromolecules such as proteins (bound forms) [49], [56]. The relative proportions of free and conjugated polyamines may vary among different plant species [50]. Therefore, all three forms were analyzed, but the conjugated and bound forms were either undetectable or represented less than 10% of the total polyamines (data not shown) and we only present the free polyamine contents in this paper.

**Figure 2:**
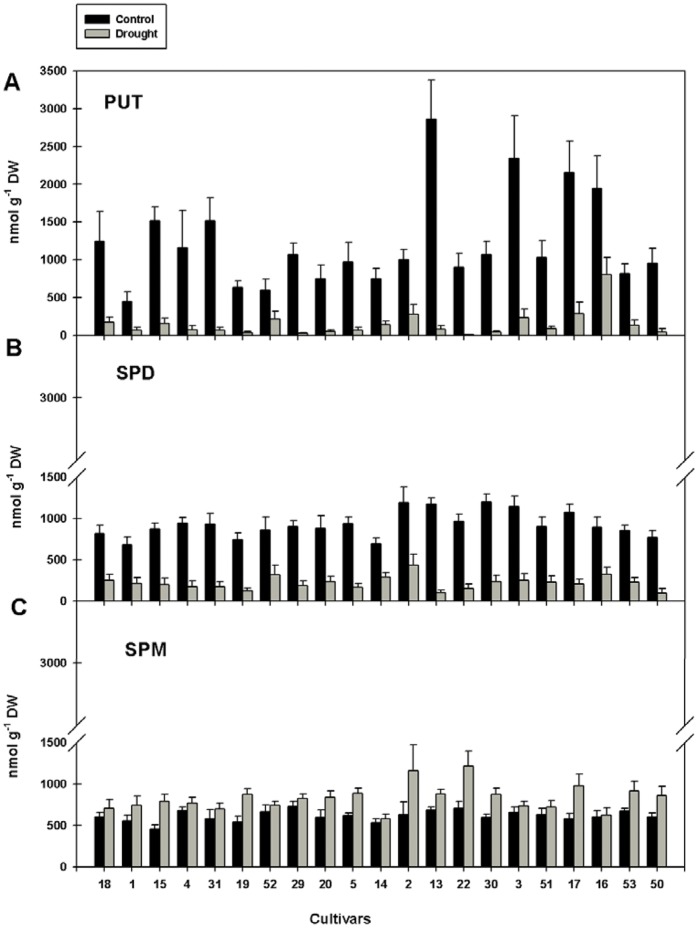
Polyamine content under control and drought conditions. The different panels show Put (A), Spd (B) and Spm (C) content of leaves of 21 rice cultivars. Cultivars numbered from 1 to 50 were sorted from most tolerant to most sensitive. Each value represents the mean (±SE) of two experiments with five replicates each.

Under control conditions, leaf Put content (448 to 2863 nmol/g DW) showed higher variation between the cultivars than Spd (678 to 1195 nmol/g DW) and Spm (461 to 727 nmol/g DW) content (Fig. 2). Some of the *japonica* cultivars (13, 3, 17 and 16) showed substantially higher Put levels already under control conditions in comparison to the other cultivars. After 18 days of drought stress, Put and Spd levels were strongly reduced (2.4 to 87.1-fold for Put and 2.4 to 11.1-fold for Spd). In contrast, Spm levels were either unchanged or slightly increased. While Put was the predominant polyamine under control conditions, Spm became predominant under drought stress but there were no significant correlations between polyamine content and drought tolerance of the cultivars.

**Figure 3:**
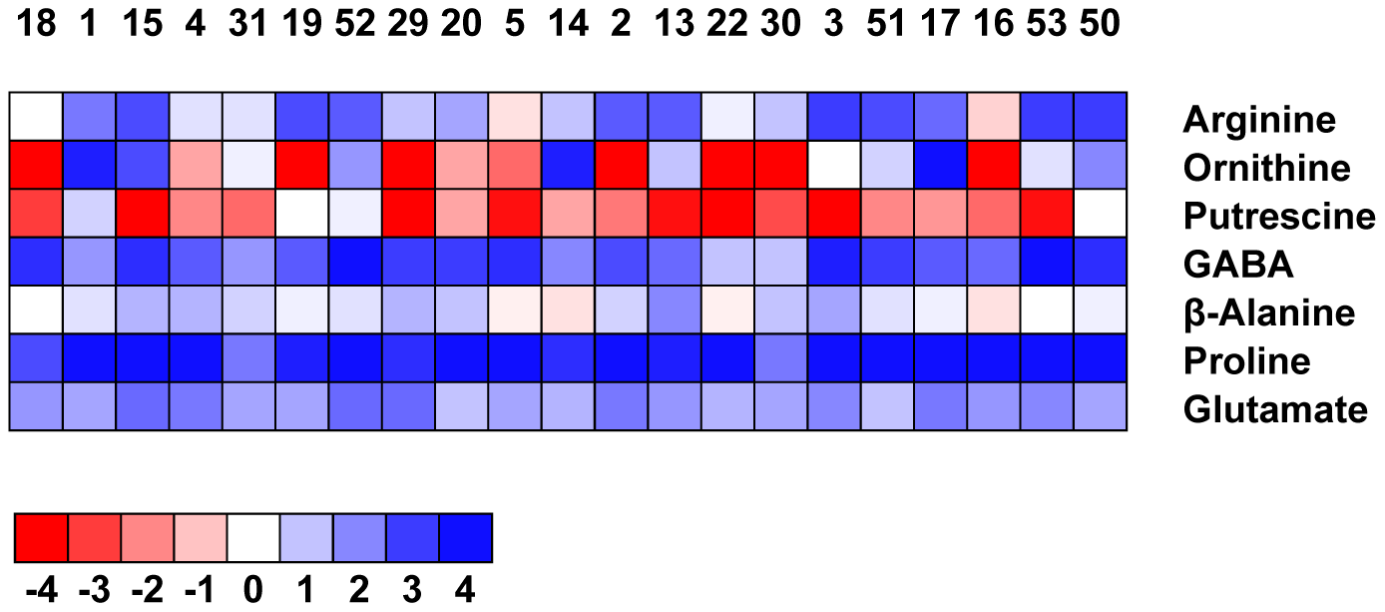
Fold change of selected metabolites under drought in comparison to control conditions. Fold change (log2) of metabolites in leaves of 21 cultivars is shown. Cultivars numbered from 1 to 50 were sorted from most tolerant to most sensitive. Data represent the means of five biological replicates from one experiment.

GC-MS measurements were used to quantify the relative pool sizes of additional metabolites related to polyamine biosynthesis and degradation (Fig. 3). Arginine and ornithine are involved in the biosynthetic pathway, while 4-aminobutyric acid (GABA), 1,3-diaminopropane and ß-alanine are polyamine degradation products. 1,3-diaminopropane, however, was not detectable under control conditions. All other metabolites related to polyamine biosynthesis and degradation are either not measurable by GC-MS or were not detectable in our samples. Additional metabolites related to polyamine metabolism are glutamic acid and proline. The latter is also synthesized from arginine and ornithine and therefore competes with the polyamine biosynthesis pathway for these substrates [57], [58].

The decrease of Put levels under drought stress that was already observed in the HPLC measurements was also evident from the GC-MS data, except for no change in cultivar 19 and 50 and a very small increase in cultivar 1 and 52 (Fig. 3). In addition, ornithine, which due the chemical derivatization required for GC-MS measurements represents the sum of the ornithine, arginine and citrulline pools, also decreased strongly in several cultivars, but stayed constant or even increased in others. Hardly any changes were detected for ß-alanine. An arginine and citrulline specific chemical derivative detectable in the GC-MS profiles indicated that levels were either unchanged under drought conditions, or even increased, depending on the cultivar. Finally, the pool sizes of proline, GABA and glutamic acid all increased under drought stress. There were no significant correlations among the levels of these metabolites and drought tolerance of the different cultivars.

### Identification of Genes Encoding Enzymes in the Polyamine Biosynthesis Pathway

To analyze the expression of genes encoding enzymes of the polyamine biosynthetic pathway, the *Oryza sativa* genome databases TIGR and Gramene were searched for the respective genes. All together 21 genes for the following enzymes were identified: arginine decarboxylase (*ADC1, 2, 3*), agmatine iminohydrolase (*AIH*), N-carbamoylputrescine amidohydrolase (*CPA1, 2, 3, 4*), ornithine decarboxylase (*ODC1, 2, 3*), S-adenosyl-L-methionine decarboxylase (*SAMDC1, 2, 3, 4, 5, 6*), spermidine synthase/spermine synthase (*SPD/SPM1, 2, 3, 4*) (Table 3).

**Table 3:** List of genes and putative genes encoding enzymes of the polyamine biosynthetic pathway in rice.

Name	Locus TIGR	Annotation TIGR
*ADC1*	LOC_Os06g04070	Pyridoxal-dependent decarboxylase, pyridoxal binding domain
*ADC2*	LOC_Os04g01690	Pyridoxal-dependent decarboxylase protein, putative, expressed
*ADC3*	LOC_Os08g33620	Arginine decarboxylase, putative
*ODC1*	LOC_Os09g37120	Pyridoxal-dependent decarboxylase protein, putative, expressed
*ODC2*	LOC_Os04g04980	Pyridoxal-dependent decarboxylase protein, putative
*ODC3*	LOC_Os02g28110	Ornithine decarboxylase, putative, expressed
*AIH*	LOC_Os04g39210	Agmatine deiminase, putative, expressed
*CPA1*	LOC_Os03g07910	Nitrilase, putative, expressed
*CPA2*	LOC_Os06g10420	Nitrilase, putative, expressed
*CPA3*	LOC_Os12g31830	Nitrilase, putative, expressed
*CPA4*	LOC_Os02g33080	N-carbamoylputrescine amidase, putative, expressed
*SPD/SPM1*	LOC_Os02g14190	Spermidine synthase, putative, expressed
*SPD/SPM2*	LOC_Os02g15550	Spermidine synthase, putative, expressed
*SPD/SPM3*	LOC_Os06g33710	Spermidine synthase, putative, expressed
*SPD/SPM4*	LOC_Os07g22600	Spermidine synthase, putative, expressed
*SAMDC1*	LOC_Os02g39795	S-adenosyl-l-methionine decarboxylase leader peptide, putative, expressed
*SAMDC2*	LOC_Os04g42095	S-adenosyl-l-methionine decarboxylase leader peptide, putative, expressed
*SAMDC3*	LOC_Os05g04990	Adenosylmethionine decarboxylase, putative, expressed
*SAMDC4*	LOC_Os09g25625	S-adenosyl-l-methionine decarboxylase leader peptide, putative, expressed
*SAMDC5*	LOC_Os05g13480	S-adenosylmethionine decarboxylase proenzyme, putative
*SAMDC6*	LOC_Os09g24600	S-adenosylmethionine decarboxylase proenzyme, putative

### Co-localization of Polyamine Biosynthesis Genes with Drought-stress Related QTL

To check whether the genes encoding enzymes involved in polyamine biosynthesis localize to genomic regions contributing to drought tolerance under field conditions, we mapped the gene loci to selected drought tolerance QTL. The location of a QTL was estimated with the help of the flanking markers and QTL longer than 5 million bases were excluded. The following drought-stress related QTL of rice were considered: osmotic adjustment capacity, leaf drying, stomatal resistance, relative water content, leaf rolling and relative growth rate (Table 3). Of the 21 investigated genes 13 fell within these QTL regions. Three of these genes (*AIH*, *SPD/SPM1*, *SPD/SPM2*) matched overlapping regions of different drought-stress related QTL and were therefore assigned to multiple QTL in Table 4.

**Table 4:** Position of genes encoding enzymes involved in polyamine biosynthesis and position of corresponding drought-stress related QTL in the rice genome.

Gene	TIGR Locus Identifier	Chr	Start Position (kbp)	End Position (kbp)	QTL-ID	QTL start (kbp)	QTL end (kbp)	Trait	Reference
*ADC3*	Os08g33620	8	20995	20995	CQAV6	17438	25593	osmotic adjustment capacity	[116]
					CQAV7				
					CQAV9				
*AIH*	Os04g39210	4	23130	23133	AQDX006	12500	24691	osmotic adjustment capacity	[117]
*AIH*	Os04g39210	4	23130	23133	AQD018	22356	24023	leaf drying	[118]
*CPA2*	Os03g07910	3	40234	4027	AQDL002	3496	12148	stomatal resistance	[119]
*ODC1*	Os09g37120	9	21410	21409	DQE53	21370	22196	relative water content	[120]
*SAMDC2*	Os02g39790	2	24041	24043	AQD017	22596	24950	leaf drying	[118]
*SAMDC3*	Os05g04990	5	2418	2417	DQE38	2091	2782	relative growth rate	[120]
*SAMDC4*	Os09g25620	9	15386	15385	DQE42	11808	15548	relative growth rate	[120]
*SAMDC5*	Os09g24600	9	14652	14651	DQE42	11808	15548	relative growth rate	[120]
*SAMDC6*	Os05g13480	5	7476	7477	CQAV8	6133	18876	osmotic adjustment capacity	[116]
*SPD/SPM1*	Os06g33710	6	19614	19618	DQE7	17680	29028	leaf drying	[120]
*SPD/SPM1*	Os06g33710	6	19614	19618	DQE52	17680	29028	relative water content	[120]
*SPD/SPM2*	Os07g22600	7	12722	12716	AQA047	6779	20733	leaf rolling	[121]
*SPD/SPM2*	Os07g22600	7	12722	12716	DQE27	10200	15800	leaf rolling	[120]
					CQAI50				[121]
*SPD/SPM2*	Os07g22600	7	12722	12716	AQD019	11361	14500	leaf drying	[118]
*SPD/SPM2*	Os07g22600	7	12722	12716	AQD015	14500	16874	leaf rolling	[118]
*SPD/SPM3*	Os02g15550	2	8730	8727	AQDX004	7707	17485	osmotic adjustment capacity	[117]
*SPD/SPM4*	Os02g14190	2	7776	7773	AQDX004	7707	17485	osmotic adjustment capacity	[117]

Chr – chromosome.

### Expression Analysis of Polyamine Biosynthesis Genes

Expression analysis for 21 genes encoding enzymes involved in polyamine biosynthesis was performed with leaf material from nine cultivars of widely differing tolerance levels. Leaf material from five plants per cultivar and treatment was pooled from two biological experiments each and three technical replicates were measured from each pooled sample. Expression of candidate genes was normalized to that of house-keeping genes. Therefore, a positive value of relative expression indicates higher expression of the respective gene compared to the housekeeping genes and a negative value represents lower expression.

**Figure 4:**
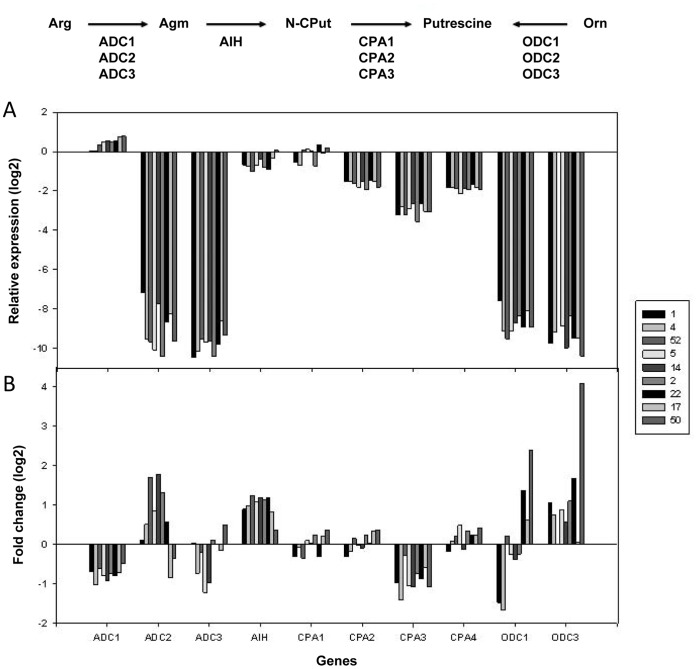
Expression of genes encoding enzymes involved in Put biosynthesis under control and drought conditions. Panel (A) shows relative gene expression (log2) under control conditions determined for nine selected cultivars. Panel (B) shows the log2 fold change values for the gene expression under drought compared to control conditions. Data represent the means of two biological experiments with three technical replicates each. Cultivars were sorted from most tolerant to most sensitive.

There are two alternative pathways to synthesize Put, either directly from ornithine, catalyzed by ODC or indirectly from arginine, catalyzed by the enzymes ADC, AIH and CPA. In total, the expression of 11 genes encoding enzymes involved in the biosynthesis of Put was well detectable and analyzed (Fig. 4), whereas the expression of *ODC2* was very low or undetectable in all samples. Under control conditions, the relative expression of the different genes averaged over all cultivars showed high variation (Fig. 4A). The expression of *ADC1* (0.45) was higher than that of *ADC2* (−9.02) and *ADC3* (−9.73) and the relative expression of *CPA1* (−0.14) was higher than that of *CPA2* (−1.63), *CPA3* (−3.01) and *CPA4* (−1.87). The relative expression of *AIH* (−0.62) was slightly lower than the expression of the house-keeping genes, whereas the expression of *ODC1* and *ODC3* was much lower (−8.70 and −9.43), but comparable to that of *ADC2* and *ADC3*. Only *ADC1* transcript abundance was correlated with drought tolerance (r = 0.865, p = 0.0026) under control conditions, where the more tolerant cultivars showed lower expression levels.

Under drought conditions, the most striking differences were a clear induction of the expression of *AIH* in all nine cultivars and of *ADC2* in six cultivars while the two most sensitive cultivars 17 and 50 showed a reduction (Fig. 4B). In the case of *ODC1*, the log2 fold change in transcript abundance under drought stress was linearly correlated with the drought tolerance of the cultivars (r = 0.916, p = 0.0005). Expression levels of *ODC3* were induced in almost all cultivars with a high induction in the sensitive cultivar 50, but did not show a significant (P<0.05) correlation with the tolerance level. In addition, reduced amounts of transcripts under drought conditions in all cultivars were observed for *ADC1* and *CPA3*, while *ADC3*, *CPA1, 2* and *4* transcript levels showed no consistent changes under drought conditions.

**Figure 5:**
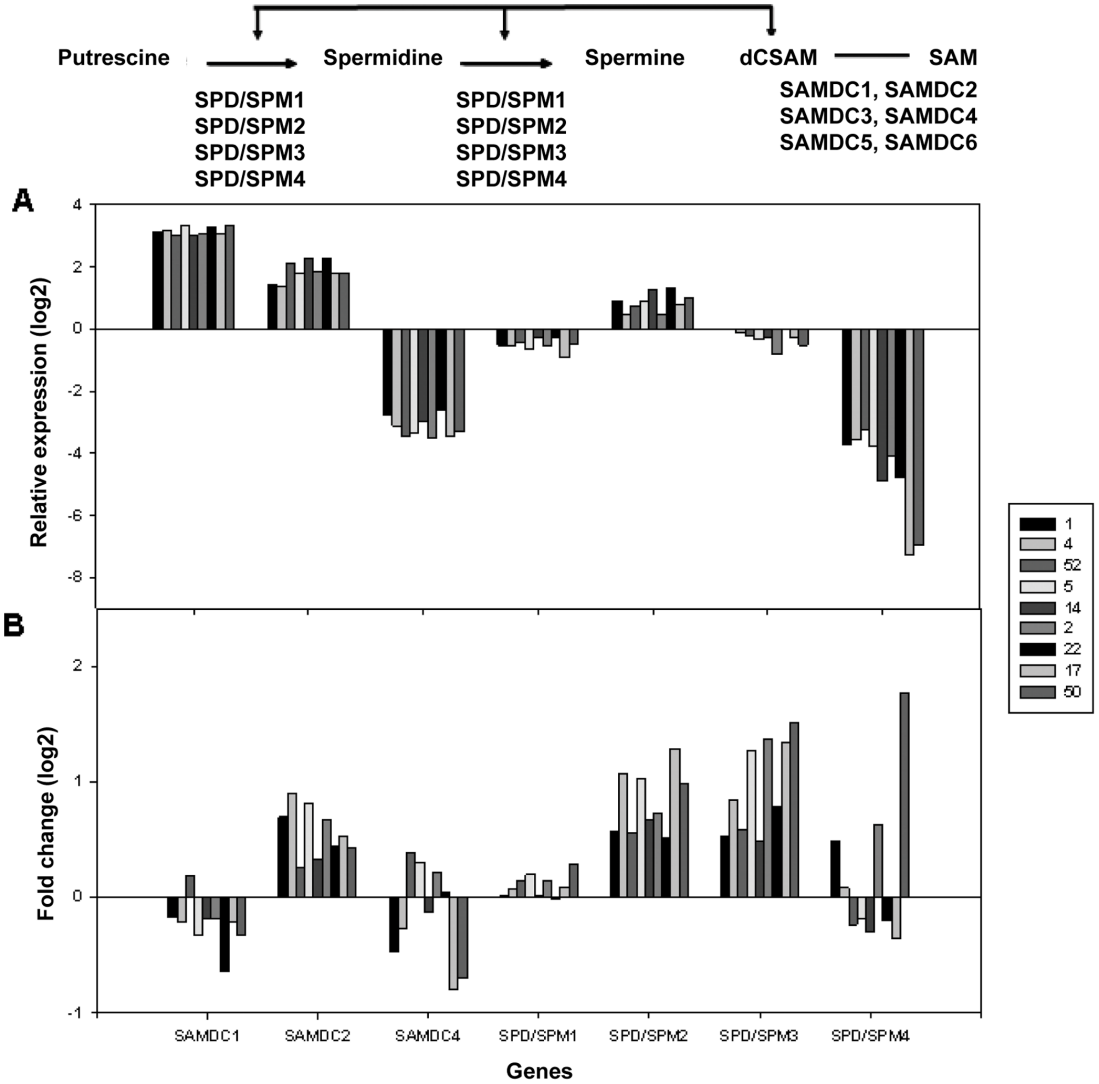
Expression of genes encoding enzymes involved in Spd/Spm biosynthesis under control and drought conditions. Panel (A) shows relative gene expression (log2) under control conditions determined for nine selected cultivars. Panel (B) shows the log2 fold change values for the gene expression under drought compared to control conditions. Data represent the means of two biological experiments with three technical replicates each. Cultivars were sorted from most tolerant to most sensitive.

Of the ten genes encoding enzymes involved in the biosynthesis of Spd and Spm, the transcripts of *SAMDC3*, *SAMDC5* and *SAMDC6* were undetectable in all samples. Under control conditions, the relative expression of *SAMDC1* (3.5) and *SAMDC2* (1.86) was higher than that of *SAMDC4* (−3.17). The relative expression of *SPD/SPM2* (0.87) was higher than that of *SPD/SPM3* (−0.28), *SPD/SPM1* (−0.51) and *SPD/SPM4* (−4.69) (Fig. 5A). The expression level of *SPD/SPM4* was especially low in the most sensitive cultivars 17 and 50 and showed a linear negative correlation with the tolerance levels of the cultivars (r = −0.86, p = 0.00297). Under drought conditions, a slight induction was observed in all cultivars for *SAMDC2* (average 0.56), *SPD/SPM2* (average 0.82) and *SPD/SPM3* (0.97), whereas the expression level of *SPD/SPM4* was only induced in three cultivars, with the highest induction in cultivar 50 with log2 1.77 (Fig. 5B). The expression of *SAMDC1* was slightly repressed in most cultivars, while the expression of *SPD/SPM1* was not consistently changed under drought stress. *SAMDC4* showed a reduction in the expression in three cultivars. No correlations could be observed between the changes in expression levels of these genes and the tolerance levels of the cultivars, except for a weak correlation (r = 0.724, p = 0.0273) for *SPD/SPM3,* which showed a higher induction in the more sensitive cultivars.

## Discussion

### Characterization of Natural Diversity under Mild Long-term Drought Stress Conditions

To elucidate the possible role of polyamine metabolism in the drought tolerance of rice, 21 cultivars were characterized under long-term drought stress conditions in the juvenile vegetative growth stage. Drought tolerance was measured by visual scoring of leaf damage which was shown before to be a reliable index for drought tolerance [2], [59], [60]. Classification of the cultivars Nipponbare and Taipei309 as drought-sensitive is in agreement with previous work [61], whereas DR2, formerly classified as drought-tolerant [62], was an intermediate cultivar under our conditions.

Water loss during drought stress caused mid-day leaf water potentials of −0.95 to −1.17 MPa for different cultivars under control and −1.77 to −2.56 MPa under drought conditions. The values under stress conditions are higher than those of Turner et al. who reported a decrease in mid-day LWP from −1.0 to −2.3 MPa for dryland and −1.8 to −2.9 MPa for wetland cultivars after 10 days of differential irrigation sufficient for induction of complete leaf rolling [63]. On the other hand, our findings are comparable with results for 40-day old rice plants exposed to a water deficit for 14 days [64] and were in between the values of LWP after 30 days of water stress under field conditions [65]. Under stress, the decrease of mid-day LWP was also similar to that in three week old rice plants subjected to a gradual water stress over 23 days in the greenhouse [66].

Drought stress significantly reduced total biomass in all cultivars by 15.8 to 36.4%, which was lower than in other moderate drought stress trials under field conditions where biomass was reduced by 45% [14], [67]. Larger plant size at the end of the drought stress is associated with superior recovery ability after stress release [13], [68]. Furthermore, biomass under stress correlates with yield [67] and is used as an index for drought tolerance.

Tolerant cultivars exhibited significantly higher WUE indicating that tolerant cultivars maintained a higher biomass using less water per dry weight. Hence, the tolerant cultivars were not just able to extract more of the available water but also used it more efficiently, reflected by a significant correlation between the drought score and the shoot dry weight. Plants with higher WUE produce more dry matter than plants with lower WUE as drought stress intensifies, as shown for the doubled-haploid line population from a cross between the cultivars IR 62266 and CT 9993 [69].

Photosynthetic rate of higher plants is known to decrease as the leaf water potential decreases [70]. Depending on the cultivars the reduction ranged from 77% to 97% in comparison to control plants on day 18 of stress treatment. Stomatal limitation is the main determinant of reduced photosynthesis under drought stress [71], although contents and activity of photosynthetic carbon reduction cycle enzymes including Rubisco are also reduced [72].

### Polyamine Metabolism-related Metabolite Content of Rice Changes during Drought Stress

It has repeatedly been observed that plants change their polyamine content under abiotic stress conditions. However, results of polyamine measurements are often contradictory and the physiological significance of the changes is often unclear although various protective mechanisms, such as membrane or nucleic acid stabilization, scavenging of free radicals and contributions to osmotic adjustment and ion homeostasis have been suggested [34], [36].

In the present study the levels of Put and Spd decreased significantly under drought stress, while Spm accumulated in all investigated cultivars, making it the most abundant polyamine under drought stress. The accumulation of Spm is in accordance with previous results in rice [39], [46], [73] and other species under drought, osmotic or salt stress [31], [45], [74]–[76]. Furthermore, transgenic rice over-expressing the *Datura stramonium ADC* gene exhibited higher drought tolerance due to the conversion of Put to Spd and Spm [32]. Rice plants expressing *SAMDC* from *D. stramonium* showed unaltered levels of Put and an increase in Spm at the expense of Spd, leading to similar drought symptoms as in the wild type, but a more robust recovery upon return to well-watered conditions [77]. An Arabidopsis mutant unable to produce Spm showed hypersensitivity to salt and drought and the phenotype could only be restored by pretreatment with Spm, but not with Put or Spd [78]. These results suggest that Spm may be beneficial for plants under drought and salt stress.

In contrast, Spd levels decreased in all cultivars under drought stress similar to barley under drought [42] and in rape seed under osmotic stress [44]. Under osmotic stress Spd accumulated in oat, wheat and rice [31], [32], [39], [45]. Also Arabidopsis plants overexpressing the *SPD* gene from *Cucurbita ficifolia* contained more Spd in their leaves and showed enhanced drought tolerance [79].

Put levels decreased in rice leaves in our drought stress experiments in accordance with results from salt stress experiments in rice [46], [80], [81] and tomato [76]. However, Put accumulated in response to drought in several other species [31], [38], [39], [41], [43], [82]. Also in Arabidopsis overexpression of the homologous *ADC2* gene conferred drought tolerance after accumulation of putrescine [83].

Bouchereau et al. [34] proposed that sensitive organisms generally accumulate Put under stress conditions and are unable to accumulate Spd and Spm, in agreement with the observation of increased Spd and Spm levels in a drought-tolerant wheat cultivar, while a drought-sensitive cultivar accumulated high levels of Put [31]. However, other reports showed the opposite behaviour e.g. in wheat [84] and alfalfa [85]. Also in the present study under controlled long-term drought stress there were no significant correlations detectable between drought tolerance and polyamine content or the ratios in the content of different polyamines.

The pool size of arginine, the main substrate for polyamine biosynthesis, increased under drought conditions, indicating that this pathway was not substrate limited. The other possible substrate, ornithine was increased in roughly half of the cultivars and decreased in the others. Arginine is also a precursor of proline biosynthesis [57], [58], where it is converted to ornithine through the activity of arginase [86]. Under stress conditions, however, the glutamate pathway is more important than the ornithine pathway for proline biosynthesis [57], [87] and glutamate levels were also increased suggesting that the observed accumulation of proline in our experiments was mainly derived from the glutamate pool. Similar increases in proline content have been observed in many plant species under various stress conditions (see [88] for a review).

Polyamine catabolism not only eliminates cellular polyamines, but enzymes and products of polyamine catabolism also contribute to important physiological processes [56], [89]. However, very little is known about polyamine catabolism under stress conditions. Under drought stress, the concentration of the polyamine degradation product ß-alanine was not changed, while GABA levels were clearly increased. GABA is a degradation product of Put and Spd via Δ1-pyrroline [50], which was not present in our samples in detectable quantities. This makes a high degradation rate of Put or Spd rather unlikely, although we cannot exclude that Δ1-pyrroline is degraded too rapidly to accumulate to detectable levels. Higher GABA levels were also found after dehydration in Arabidopsis [90], but GABA levels can also be influenced by other metabolic pathways [91].

We found 21 genes encoding enzymes involved in polyamine biosynthesis in the rice genome. For 17 of these genes we found evidence for expression (*ADC1, 2* and *3*, *AIH*, *CPA1, 2, 3* and 4, *ODC1,* and *3*, *SAMDC1, 2,* and *4*, *SPD/SPM1, 2, 3* and *4*). Under control conditions the relative expression of *ADC1*, *AIH* and *CPA1* was higher than the expression of *ADC2* and *3*, *CPA2, 3* and *4* and *ODC1* and *3*, suggesting an important role of *ADC1*, *AIH* and *CPA1* for the normal growth of plants. This is consistent with reports that the ADC pathway is predominant in higher plants [92], [93]. However, also a similar expression for an *ADC* and an *ODC* gene under control conditions in rice was shown [94]. Lower expression of *ADC2* than *ADC1* was previously reported [95] as well as a low expression of *ODC* [96]–[98].

When expression under control and drought conditions was compared, the genes could be divided into four groups: constitutively expressed (*CPA1, 2, 4, SAMDC1, 4, SPD/SPM1*), drought induced (*ADC*2, *AIH, ODC3, SAMDC2*, *SPD/SPM*2, 3), drought repressed (*ADC1, CPA3*) and genes with cultivar specific responses (*ADC3, ODC1, SAMDC4, SPD/SPM4*). This is in agreement with a recent microarray study using the cultivars 50 and 52, covering nine of the investigated genes [99]. In Arabidopsis the genes *ADC2*, *SPDS1* and *SPMS* responded most under drought stress [52]. The expression of three genes showed a correlation with the drought tolerance of the cultivars. *ADC1* was already more highly expressed, while *SPD/SPM4* showed lower expression in sensitive cultivars under control conditions. In contrast, *ODC1* showed a higher induction of its expression under drought conditions in sensitive than in tolerant cultivars. However, since we did not observe any corresponding correlations in polyamine content or ratios the physiological significance of these correlations is not obvious.

ADC is thought to be the enzyme primarily responsible for abiotic stress-induced Put accumulation [49], [100]. In response to drought stress, the expression level of *ADC2* was up-regulated, with the exception of the two most sensitive cultivars. This is in accordance with observations in Arabidopsis [53], [101] and in mustard [47], where also only *ADC2* is up-regulated in response to osmotic, drought and salt stress, respectively. Induction of *SAMDC* has been shown under drought conditions in rice [102]–[105] and under salt stress in wheat [106]. However, the induced *SAMDC* gene identified by Li and Chen in rice is homologous to *SAMDC1*, which was not induced by stress in the present study [102].

### A Model of the Drought Response of Polyamine Metabolism

**Figure 6:**
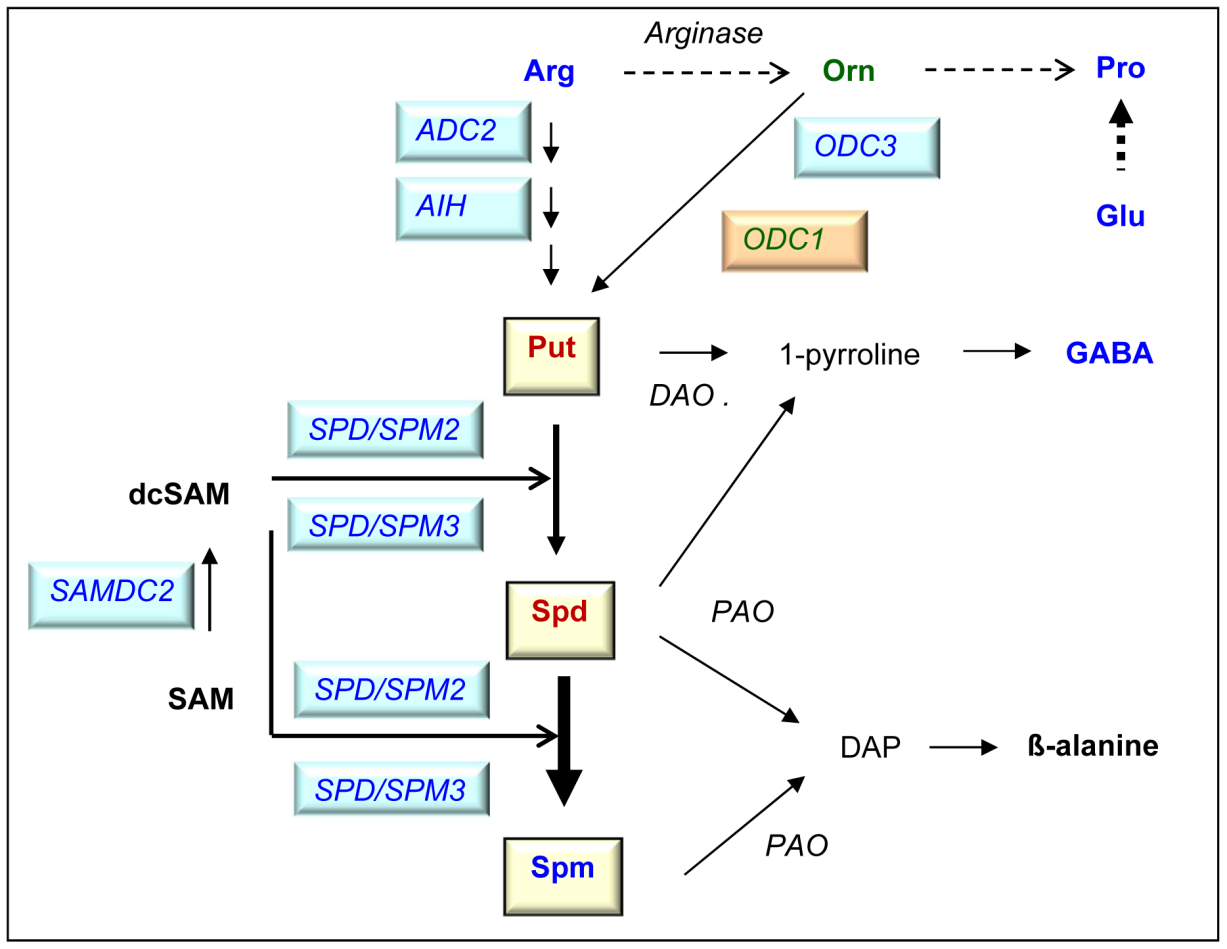
Model for the regulation of polyamine metabolism in response to drought stress in rice leaves. Red - decrease, blue – increase, green – cultivar dependent, black – not measured. Arg, arginine; Orn, ornithine; SAM, S-adenosylmethionine; dcSAM, decarboxylated S-adenosylmethionine.

Combining the metabolite analyses with an analysis of the expression of all genes encoding enzymes involved in polyamine biosynthesis allowed us to propose a model for the regulation of polyamine metabolism in response to drought stress in rice (Fig. 6). Arginine and for some cultivars ornithine are used to produce Put via the ADC and/or ODC pathways, which are transcriptionally up-regulated as indicated by the increased expression levels of *ADC*2, *AIH*, *ODC3* and for some cultivars *ODC1* under drought. The expression of *CPA3,* encoding N-carbamoylputrescine amidohydrolase also involved in Put biosynthesis, was down-regulated under drought conditions. This was potentially compensated by the constitutive expression of three other *CPA* genes, *CPA1, 2* and *4.* Put is not accumulated, but is converted into Spd and Spm by the addition of aminopropyl groups from dcSAM by SAMDC and SPD/SPM. On the transcript level, this is supported by the increased expression levels of *SPD/SPM2* and *3*. Conversion of SAM to dcSAM, the necessary substrate for these reactions, seems to be also increased as indicated by an increase in the transcript level of *SAMDC2*. This might lead to efficient conversion resulting in decreased Put and Spd levels and the accumulation of Spm. Nevertheless, to further verify this model, measurements of enzyme activities and consideration of Put and Spd degradation will be necessary. Also Alcazar et al. described a Put to Spm canalization in response to drought in *Arabidopsis thaliana* and *Craterostigma plantagineum* but only observed an increase of Spd and Spm in *C. plantagineum* [107]. Whether the increased GABA levels under drought stress were the result of polyamine degradation could not be decided on the basis of the current data.

Of the expressed genes related to polyamine biosynthesis, 11 were localized in QTL for traits related to drought tolerance of rice. Also, all stress inducible genes, except for *ADC2, ODC3* and *ODC1* that is only induced in sensitive cultivars, are located in these QTL. This suggests that polyamine metabolism may contribute to the differential drought tolerance of rice cultivars under field conditions, although no simple correlations could be found.

## Materials and Methods

### Plant Material, Cultivation and Drought Stress Treatment

Twenty-one rice (*Oryza sativa* L.) cultivars originating either from the IBT (Institute of Biotechnology, Hanoi, Vietnam) or from the IRRI (International Rice Research Institute, Manila, Philippines) were grown under water sufficient and water limiting conditions in three independent experiments in a controlled climate chamber as described before [99]. Plants were cultivated in 10 cm pots on a 7.5 cm deep layer of an artificial substrate. Pots were positioned in polypropylene boxes filled with water to the level of the substrate surface. Rice plants were grown in 12 h days (600 µE m^−2^ s^−1^) at 26°C and 75% relative humidity and 12 h nights at 22°C and 70% relative humidity. Twenty-six days after sowing, water was removed from half of the boxes and plants were left to dry for four days, until the soil water content had reached the permanent wilting point (PWP) for 50% of the plants. Thereafter, the soil water content was kept constant to the fixed PWP value over a period of 14 days by weighing each pot at the end of the light period and adding the amount of water lost during the last 24 hours.

### Physiological Characterization of the Plants, Sampling and Statistics

The leaf phenotype was visually assessed for individual plants before and during drought stress treatment (on day 0, 4, 11 and 18 of stress) based on the stress damage score of the IRRI [108], in which ‘1′ represents plants with undamaged leaves, ‘9′ almost or completely dead plants. The number of tillers was counted and the plant height [109] was measured at the same time. Chlorophyll-*a* fluorescence and leaf temperature were measured with a pulse-amplitude-modulated Dual- PAM-100 fluorometer (WALZ, Effeltrich, Germany) on the middle section of a second fully expanded leaf during mid-day without dark adaptation and under climate chamber conditions. The effective quantum yield of PS II (ΔF/Fm’) was determined from the maximum light-adapted fluorescence yield (Fm’) and the current fluorescence yield (Ft) as (ΔF/Fm’ = (Fm’ - Ft)/Fm’). Pre-dawn and midday leaf water potential was measured after 18 d and 24 d of treatment, respectively, with a Scholander pressure bomb (Soil Moisture Equipment Corporation, Santa Barbara, CA, USA). After 18 d of drought stress, plants were harvested four to six hours after the beginning of the light period. Fully expanded green leaf blades were harvested for expression analysis by quantitative RT-PCR (qRT-PCR) and polyamine and metabolite analysis and immediately frozen in liquid nitrogen. The remaining plants was harvested completely to determine fresh weight (FW) and dry weight (DW, 48 h, 80°C) of leaf blades, total shoots and roots.

Daily water use of the plants was calculated as pot mass after addition of water minus pot mass after 24 h evapotranspiration (prior to addition of water) for each day during the drought period. Water use efficiency (WUE) was then calculated as the average daily water use divided by dry biomass of the plant at harvest.

Performance parameters were evaluated with Statistical Analysis Software (SAS 9.2, SAS-Institute, Cary, NC, USA). Drought score values were ranked and the average rank was calculated within each experiment. Results of three experiments were combined and mean and standard deviation of the average ranks was calculated for every cultivar. The relative dry weight of the shoot and the entire plant were calculated by dividing the average shoot or plant dry weight under drought condition for each cultivar and experiment by the respective control value. The effects of experiment, cultivar, condition and the interaction of condition x cultivar on PAM yield, FW and DW of the shoot and the entire plant were estimated with SAS general linear model procedure (PROC GLM) (Sum of squares type III, degrees of freedom (df) (condition) = 1.58; df (cultivar) = 20.58). The effect of experiment and cultivar were tested with PROC GLM for those parameters that were only measured under drought conditions (Score, WUE and relative dry weight, df (cultivar) = 20, 28). The Spearman correlation between the negative rank of the drought score and all performance parameters was determined with PROC CORR, Spearman (n = 51).

### Polyamine Analysis

Polyamines (Put, Spd and Spm) were quantified by High Performance Liquid Chromatography (HPLC) using the dansylation method described by Smith and Davies [110]. Leaf samples (100–200 mg) were homogenized using a ball mill (Retsch, Haan, Germany) and extracted in 1 ml of 0.2 N perchloric acid (PCA) for 1 h at 4°C. After centrifugation at 16000×g at 4°C for 30 min, the supernatant and pellet were collected separately. The supernatant was used to determine free polyamines. To extract PCA-soluble conjugated polyamines, 200 µl of the supernatant were hydrolyzed with 200 µl of 12 N HCl at 110°C for 18 h. Afterwards, HCl was removed at 70°C and polyamines were re-suspended in 200 µl of 0.2 N PCA. To extract PCA insoluble bound polyamines, the pellets of the first extraction were rinsed twice with 1 ml of 0.2 N PCA to remove soluble polyamines, dissolved in 200 µl of 1 N NaOH and sonicated for 90 min. Then acid hydrolysis was performed as described above.

Polyamines were derivatized with dansyl chloride [110]. Ten µl of 0.5 mM diamino-hexane as an internal standard were added to 100 µl aliquots of each fraction and then 110 µl of 1.5 M sodium carbonate and 200 µl dansyl chloride (7.5 mg/ml in acetone) (Sigma, Munich, Germany) were added. After 1 h incubation at 60°C in the dark 50 µl of a 100 mg/ml proline solution were added to bind free dansyl chloride. After 30 min incubation at 60°C in the dark, dansylated polyamines were extracted with 250 µl toluene, dried in a vacuum centrifuge and dissolved in 100 µl methanol.

HPLC analysis was performed with a reverse phase LC-18 column (Supelco, Munich, Germany) on a system (Dionex, Germering, Germany) consisting of a gradient pump (model P 580), an automated sample injector (ASI-100) and a fluorescence detector (RF 2000). Twenty µl samples were injected and polyamines were eluted with a linear gradient from 70% to 100% (v/v) methanol in water at a flow rate of 1 ml/min. Dansylated polyamines were detected at an excitation wavelength of 365 nm and an emission wavelength of 510 nm. Data were analyzed using the Chromeleon software (Dionex, Germering, Germany) and calibration curves obtained from the pure substances.

### Metabolite Profiling

Leaf samples (120 mg) were shock frozen in liquid nitrogen and a fraction enriched in polar primary metabolites was prepared and processed as described previously [81]. Gas chromatography coupled to electron impact ionization-time of flight-mass spectrometry (GC/EI-TOF-MS) was performed using an Agilent 6890N24 gas chromatograph hyphenated to a Pegasus III time-of-flight mass spectrometer, LECO, St. Joseph, USA [111]. Chromatograms were acquired and processed by CHROMATOF software 1.00, Pegasus driver 1.61 (Leco; http://www.leco.de). Selective peak heights representing arbitrary mass spectral ion currents were normalized by sample dry weight and to an internal standard which was added upon extraction of the polar metabolite fraction. GC-TOF-MS chromatography data processing was performed using the TagFinder software [112]. Metabolites were identified under manual supervision using the TagFinder, the NIST08 software (http://chemdata.nist.gov/) and the mass spectral and retention time index (RI) reference collection of the Golm Metabolome Database [113], [114].

### Quantitative RT-PCR (qRT-PCR)

Leaf material of 5 replicates per cultivar and treatment was homogenized using a ball mill and equal fractions were pooled to reach 60 mg. Total RNA was extracted using the NucleoSpin RNA plant kit (Macherey-Nagel, Düren, Germany). RNA concentration was determined photometrically (NanoDrop ND-1000 UV-Vis spectrophotometer, Nanodrop Technologies, Wilmington, DE). Purified RNA was treated with DNase I (Roche, Mannheim, Germany) and the absence of genomic DNA was confirmed by qRT-PCR, using primers for an intron within a Helix-loop-helix DNA-binding domain containing gene sequence (Os01g01840). The integrity of the final RNA samples was checked on 1.7% (w/v) agarose gels. 4 µg of total RNA were transcribed into cDNA using Superscript III Reverse Transcriptase (Invitrogen, Karlsruhe, Germany). The quality of the cDNA was checked by qRT-PCR using primers for the 5′and 3′ ends of the actin-1 (Os03g50890) and cyclophilin (Os08g19610) genes.

qRT-PCR was performed with the ABI Prism 7900HT (Applied Biosystems, Foster City, CA) in 10 µl reaction volume (1 µl cDNA, 4 µl primer mix (0.5 µM each), 5 µl SYBR Green Master Mix (Eurogentec, Köln, Germany). Genes encoding enzymes involved in polyamine biosynthesis in rice were searched in the TIGR Rice Genome Annotation (http://rice.plantbiology.msu.edu/cgi-bin/gbrowse/rice/) and Gramene ( http://www.gramene.org ) databases. Primers for qRT-PCR were designed using the software PrimerExpress (Version 2.0, Applied Biosystems) and all primer sequences are given in Table S1. Primer sequences were blasted on the Gramene and Beijing Genomics Institute (http://rice.genomics.org.cn) databases. Correct size of the amplified region for each primer pair was checked by agarose gel electrophoresis.

Data were analyzed using the SDS 2.0 software (Applied Biosystems) and normalized based on the expression data of the housekeeping genes actin 1 and cyclophilin. Normalized expression of the genes of interest was calculated by dividing the average relative expression (primer efficiency P to the power of cycle number Ct) of the two housekeeping genes (H1 and H2) by the relative expression of the gene of interest (GOI): ((P_H1_∧Ct_H1_+ P_H2_∧Ct_H2_)/2)/P_GOI_∧Ct_GOI_ [99]. Primer efficiency was calculated using LinRegPCR [115]. Fold change was calculated as log2 of the ratio of relative expression of genes under stress conditions to relative expression of genes under control conditions.

### Comparison to Quantitative Trait Loci (QTL)

The comparison of the genome position of genes encoding enzymes involved in polyamine biosynthesis with those of published drought-related QTL of rice in the Gramene database was performed as described before [99]. Genes were considered to map to QTL regions when the midpoint of the mapping coordinates of the start and end positions of the corresponding gene fell within the QTL region boundaries.

## Supporting Information

Figure S1 **Phenotype of rice plants under stress conditions in comparison to control.** 44 day old rice plants (different cultivars, randomized design) are shown under control conditions (A) and after 18 days of moderate long-term drought stress (B).
(TIF)

Table S1 **List of primers used for qRT-PCR.**
(DOCX)
