# Supplementary figures and images for: Dissecting Rice Polyamine Metabolism under Controlled Long-Term Drought Stress
Source: PLoS One. 2013 Apr 8;8(4):e60325. doi: 10.1371/journal.pone.0060325 (PMC3620119; doi:10.1371/journal.pone.0060325)

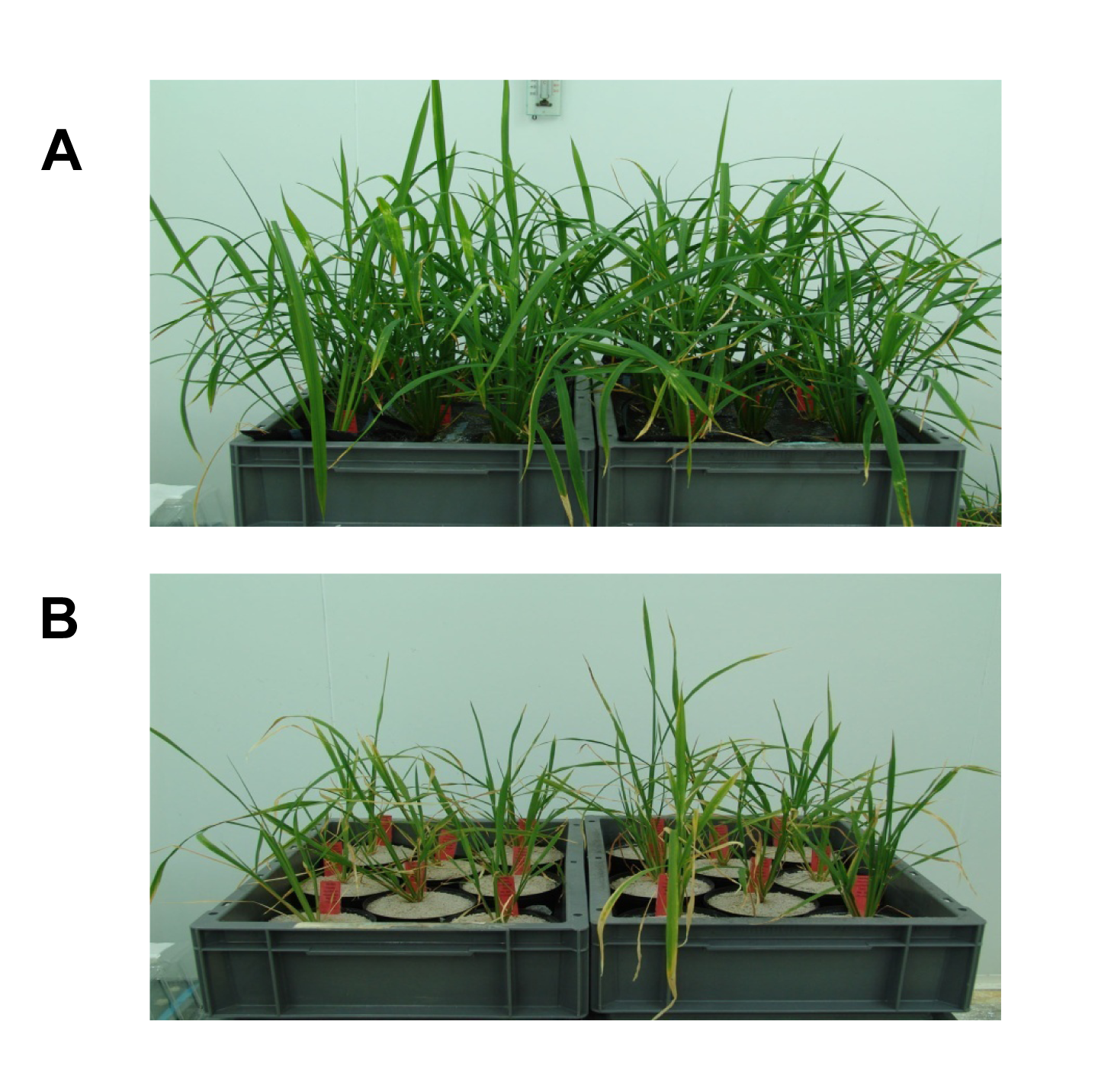

Supplement: Figure S1 — Phenotype of rice plants under stress conditions in comparison to control. 44 day old rice plants (different cultivars, randomized design) are shown under control conditions (A) and after 18 days of moderate long-term drought stress (B). (TIF) [file pone.0060325.s001.tif]
