# Supplementary material for: Dissecting Rice Polyamine Metabolism under Controlled Long-Term Drought Stress
Source: PLoS One. 2013 Apr 8;8(4):e60325. doi: 10.1371/journal.pone.0060325 (PMC3620119; doi:10.1371/journal.pone.0060325)
Supplement: Table S1 — List of primers used for qRT-PCR. (DOCX) [file pone.0060325.s002.docx]

**Table S1.** List of primers used for qRT-PCR.

| **Locus ID** | **Gene** | **Function** |  | **Primer sequence** |
| --- | --- | --- | --- | --- |
| [LOC_Os06g04070](http://www.tigr.org/tigr-scripts/euk_manatee/shared/ORF_infopage.cgi?db=osa1&orf=LOC_Os04g01690) | ADC1 | Arginine decarboxylase | FW | CGTCATCGACGTTGGTGGA |
|  |  |  | RW | CCAAGCTGTATGCCACGGAC |
| [LOC_Os04g01690](http://www.tigr.org/tigr-scripts/euk_manatee/shared/ORF_infopage.cgi?db=osa1&orf=LOC_Os04g01690) | ADC2 | Arginine decarboxylase | FW | AGAAGGTTGCGACGGAGAATG |
|  |  |  | RW | TGGTCAGCCCTTTCTTCATCA |
| LOC_Os08g33620 | ADC3 | Arginine decarboxylase | FW | AATCATCCCAATCCAGTGCCTT |
|  |  |  | RW | TGCCTCCCGCTGATGAAGT |
| LOC_Os09g37120 | ODC1 | Ornithine decarboxylase | FW | CGGCTGGCTCCAACTTCAA |
|  |  |  | RW | TGGAGTATGCCAGGTGGATCTT |
| [LOC_Os04g04980](http://rice.plantbiology.msu.edu/cgi-bin/ORF_infopage.cgi?orf=13104.m00419) | ODC2 | Ornithine decarboxylase | FW | GCTTGTGTTCGACGACATGG |
|  |  |  | RW | TGGAGTATGCCAGGTAGGTGTTTA |
| [LOC_Os02g28110](http://rice.plantbiology.msu.edu/cgi-bin/ORF_infopage.cgi?orf=13102.m03065) | ODC3 | Ornithine decarboxylase | FW | TCTCCACGTCCAACATGAAGAC |
|  |  |  | RW | GGCACTTTCCCAGTGATCTAGC |
| LOC_Os04g39210 | AIH | Agmatine iminohydrolase | FW | GCTGCTTCATTAAACCTGGGG |
|  |  |  | RW | GCATCAACGGACTTGGAGAG |
| LOC_Os02g33080 | CPA1 | N-carbomoylputrescine amidohydrolase | FW | GTCAGCTTTTTTGAGGAGGCG |
|  |  |  | RW | TACAGGCCAAGATCAGTGCCA |
| LOC_Os03g07910 | CPA2 | N-carbomoylputrescine amidohydrolase | FW | TTGTATGCTGCGAGAGGTGCT |
|  |  |  | RW | TGCAATGGCCCTGTAGTCATG |
| LOC_Os06g10420 | CPA3 | N-carbomoylputrescine amidohydrolase | FW | TGCGTTTAACATGAGCACCG |
|  |  |  | RW | TGATTGTCAACAGCCCTGGA |
| LOC_Os12g31830 | CPA4 | N-carbomoylputrescine amidohydrolase | FW | CAAAAGTTGAGGCTGTGCGAA |
|  |  |  | RW | TCCAGACGCTGTCAAACTTCC |
| [LOC_Os04g42095](http://rice.plantbiology.msu.edu/cgi-bin/ORF_infopage.cgi?orf=13104.m29426) | SAMDC1 | S-adenosylmethionine decarboxylase | FW | TGGTGACGATTCGCTGCTTT |
|  |  |  | RW | GGCTGGCTGCTTCACAGAATAA |
| [LOC_Os02g39795](http://rice.plantbiology.msu.edu/cgi-bin/ORF_infopage.cgi?orf=13102.m29135) | SAMDC2 | S-adenosylmethionine decarboxylase | FW | TTGGAGATCCTGCAAAGCCA |
|  |  |  | RW | GCCCAGTCATGCACATCTCAA |
| LOC_Os05g04990 | SAMDC3 | S-adenosylmethionine decarboxylase | FW | GTGGTGGACGAGAATGACCC |
|  |  |  | RW | TTCATGGAGTAGCCGCACG |
| [LOC_Os09g25625](http://rice.plantbiology.msu.edu/cgi-bin/ORF_infopage.cgi?orf=13109.m18569) | SAMDC4 | S-adenosylmethionine decarboxylase | FW | TGCCCTTCAGACTTTTCGGTC |
|  |  |  | RW | TCTGCACCTTTTGCCCATG |
| LOC_Os09g24600 | SAMDC5 | S-adenosylmethionine decarboxylase | FW | TGACTTTGCCGACGAGGTG |
|  |  |  | RW | GCGGAAGAGTAGGACCTCACAG |
| LOC_Os05g13480 | SAMDC6 | S-adenosylmethionine decarboxylase | FW | GTCATCCAAGTCCACCTTCTCG |
|  |  |  | RW | CATAGGCACGGTCTCCCCTTAT |
| LOC_Os06g33710 | SPD/  SPM1 | Spermidine/spermine synthase | FW | GAGCTGTGCATTATGCCTGGA |
|  |  |  | RW | GCTGGACCTTCTTTCGCACAT |
| LOC_Os07g22600 | SPD/  SPM2 | Spermidine/spermine synthase | FW | ATGGCGTGGCCTTTTTGAA |
|  |  |  | RW | TTGAGCCGGTCCTATTGGATC |
| LOC_Os02g15550 | SPD/  SPM 3 | Spermidine/spermine synthase | FW | AGAGCATGTGGTTGCATACGC |
|  |  |  | RW | AACCCTTGAATGTCTCACGGC |
| LOC_Os02g14190 | SPD/  SPM4 | Spermidine/spermine synthase | FW | CACCCTCAGGCATGTCTTCAA |
|  |  |  | RW | TGTCAGCAAAGGATGGCACA |
| LOC_Os01g01840 | Intron |  | FW | TAGAGAGTTCGATCTTGCGCG |
|  |  |  | RW | CGGCCCATTCAATGAAGTCTT |
| LOC_Os03g50890 | Actin | Actin | FW | CTCCCCCATGCTATCCTTCG |
|  |  |  | RW | TGAATGAGTAACCACGCTCCG |
| LOC_Os08g19610 | Cyclophilin | Cyclophilin | FW | CCACCATCACAGATCGGATCTT |
|  |  |  | RW | GCGGTCAGAGCGAAAGTAGCTA |
| LOC_Os03g50890 | 5´actin | Actin | FW | TACCCCATCGAGCATGGTATC |
|  |  |  | RW | TTCTCACGATTGGCCTTGG |
| LOC_Os03g50890 | 3´actin | Actin | FW | TGGAGGATCCATCTTGGCAT |
|  |  |  | RW | TCGTACTCAGCCTTGGCAATC |
